# Genomic prospecting and biochemical characterization of a novel thermostable 3-quinuclidinone reductase from hot spring metagenomes for efficient biocatalysis

**DOI:** 10.1128/aem.01490-26

**Published:** 2026-09-16

**Authors:** Deshuai Lou, Jinghao Cui, Jingfa Duan, Bingpu Zhou, Yue Wang, Dong Wang, Jun Tan, Hongtao Duan

**Affiliations:** 1 Chongqing Key Laboratory of Functional Substances from Distinctive Medicinal Plants in the Three Gorges Reservoir Area, School of Biological and Chemical Engineering, Chongqing University of Education 177518, Chongqing, China; 2 School of Information Science and Engineering, University of Jinan 12413, Jinan, China; 3 Shandong Key Laboratory of Ubiquitous Intelligent Computing, University of Jinan 12413, Jinan, China; 4 School of Medicine, South China University of Technology 506909, Guangzhou, China; Colorado School of Mines, Golden, Colorado, USA

**Keywords:** hot spring microorganisms, metagenomics, computational design

## Abstract

This study presents the discovery and characterization of a novel thermophilic 3-quinuclidinone reductase (ScQR) identified through metagenomic mining of hot spring environments. ScQR, a member of the short-chain dehydrogenase/reductase (SDR) superfamily, was heterologously expressed in *Escherichia coli*, and its catalytic properties were systematically characterized. The enzyme demonstrates exceptional thermal stability, retaining 86% of its activity after 48 hours at 70°C. Furthermore, K^+^ and Mg²^+^ ions significantly enhanced ScQR’s activity at specific concentrations. Structural analysis revealed that ScQR adopts a typical SDR fold with a conserved catalytic triad (S141-Y155-K159), and it is NAD(H) dependent. Enzyme assays indicated that ScQR is highly stereoselective for (*R*)-3-quinuclidinol, with no activity against its enantiomer, (*S*)-3-quinuclidinol. The enzyme exhibits optimal activity at pH 9 and 85°C, making it a promising candidate for industrial applications requiring high thermal stability. Molecular dynamics simulations further revealed that ScQR preserves global structural integrity up to 360 K, whereas higher temperatures induce destabilization, predominantly in the C-terminal region and residues 95–100. In addition, structure-guided computational design enabled by LigandMPNN and UniKP yielded three ScQR variants with improved substrate affinity and catalytic efficiency while maintaining the overall fold and function. This work underscores the power of metagenomics with structure-driven protein design in discovering novel enzymes with unique catalytic properties from extreme environments and establishes ScQR as a promising biocatalyst for biotechnological and pharmaceutical applications.

**IMPORTANCE:**

This study reports the discovery of ScQR, a novel thermophilic 3-quinuclidinone reductase identified via metagenomic mining. ScQR represents one of the most heat-resistant members of the SDR superfamily discovered to date, maintaining 86% activity after 48 hours at 70°C. These findings establish ScQR as a robust biocatalyst for high-temperature pharmaceutical applications and demonstrate a scalable workflow for optimizing enzymes from extreme environments, offering significant value to the fields of biocatalysis and protein engineering.

## INTRODUCTION

Most microorganisms in nature remain uncultivable under standard laboratory conditions (1). The rapid growth of large-scale sequencing data sets has propelled metagenomics into a central approach for identifying novel functional genes, especially those originating from extremophilic archaea (2, 3). These organisms constitute a largely untapped genetic reservoir, often encoding enzymes with exceptional stability and catalytic properties. Such discoveries have significantly accelerated enzyme mining efforts and contributed to the advancement of synthetic biology and biopharmaceutical development (4). Owing to their high chemical, regio-, and stereospecificity, enzymes serve as essential catalytic “building blocks” in synthetic biology (5).

**Fig 1:**
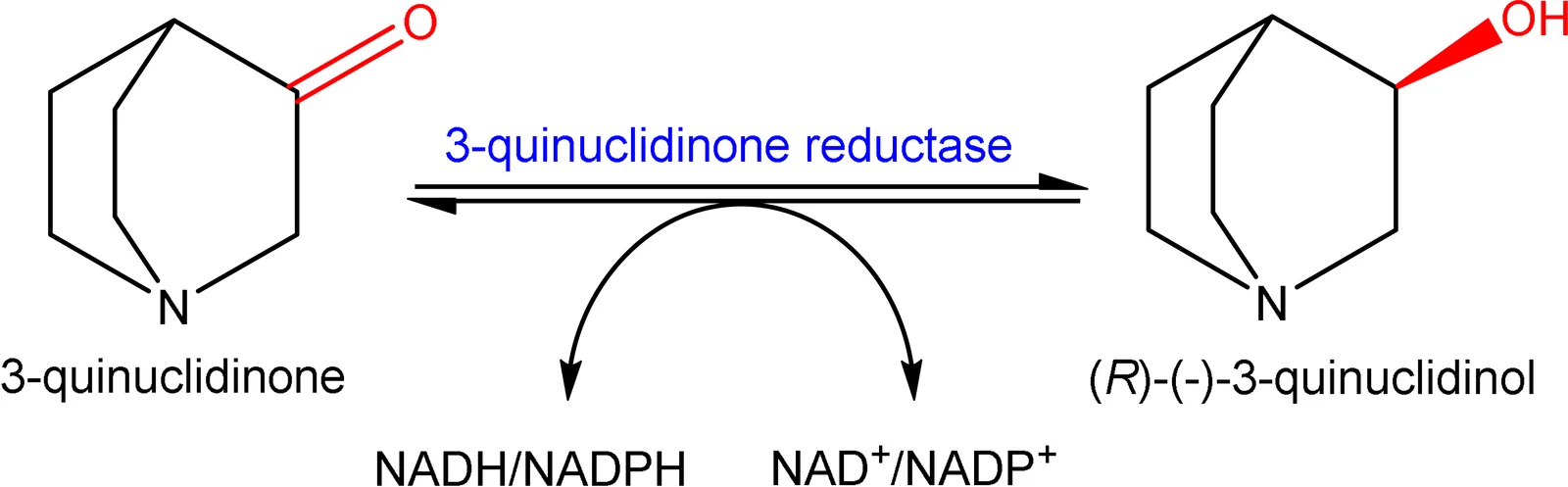
Biosynthesis of (*R*)-3-quinuclidinol mediated by 3-quinuclidinone reductase. In this process, the involvement of coenzyme I or coenzyme II ensures the proper completion of the redox reaction.

The short-chain dehydrogenase/reductase (SDR) superfamily represents a highly diverse and evolutionarily conserved group of enzymes found across all domains of life (6). SDRs participate in a wide range of fundamental metabolic processes, including the metabolism of steroid hormones, lipids, vitamins, sugars, and carbon sources (7). One member of this family, 3-quinuclidinone reductase (QR, EC 1.1.1.B51), catalyzes the stereoselective reduction of 3-quinuclidinone (CAS 3731-38-2), also known as (3*R*)-1-azabicyclo[2.2.2]octan-3-one, to (*R*)-3-quinuclidinol (CAS 25333-42-0) (Fig. 1) (8–10). (*R*)-3-Quinuclidinol is an important chiral intermediate in the synthesis of several pharmaceutical compounds, including revatropate, solifenacin, and talsaclidine (11–14). Notably, although the biosynthesis and metabolism of (*R*)-3-quinuclidinol have not been observed in living organisms, 3-quinuclidinone reductases have been identified in a number of prokaryotic and eukaryotic microorganisms (15–19). Representative examples include *Microbacterium luteolum* (accession number BAM76232.1), *Agrobacterium tumefaciens* (accession number BAR64668.1), *Ochrobactrum grignonense* (accession number OYR08778.1), and *Rhodotorula mucilaginosa* (accession number BAH28833.1). Organisms are classified into three domains: Archaea, Bacteria, and Eukarya. SDRs from each domain exhibit distinct catalytic properties. For example, SDR enzymes from archaea living in high-temperature environments, such as hot springs, are often highly thermally stable, making them valuable for biopharmaceutical applications (20). Furthermore, SDR enzymes show varying responses to metal ions, likely due to differences in charge distribution (21). Discovering novel QR in archaea can advance their use as key building blocks in synthetic biology.

As mentioned above, the exploration of microbial resources from extreme environments—particularly DNA-encoded enzymes with unique catalytic functions—frequently relies on metagenomic approaches. Through the analysis of hot spring metagenomes, a novel thermophilic 3-quinuclidinone reductase, designated ScQR (accession number UMF44229), was successfully identified. In this study, ScQR was heterologously expressed in *Escherichia coli*, and its catalytic properties were systematically characterized. Additionally, the three-dimensional structure of ScQR was predicted and analyzed, and the effects of various environmental factors on its enzymatic activity were evaluated.

## MATERIALS AND METHODS

### Chemicals and materials

The plasmid pGEX-6p-1 used for heterologous expression of ScQR was purchased from Sigma-Aldrich (USA). GSTSep Glutathione Agarose Resin for affinity chromatography and the bicinchoninic acid (BCA) protein assay kit were obtained from Yeasen Biotech (China). All DNA coding sequences were synthesized and verified by Sangon Biotech (China). *E. coli* competent cells for protein expression and other chemical reagents were supplied by Zewu Biotech (China).

### Gene discovery and heterologous expression

Metagenomic sequencing data from hot spring environments with relatively high sequencing depth were retrieved from the Sequence Read Archive database (accession number SRR10095340) in NCBI and assembled by Majorbio (China). A previously identified QR (accession number OYR08778.1) was used as a bait protein to perform BLASTP analysis against the assembled data sets. Open reading frames (ORFs) with ≥25% sequence identity to the bait were selected to construct a preliminary candidate library. A sequence similarity network was generated using the EFI-EST and visualized in Cytoscape (v3.7.1). The DNA coding sequence is fully synthesized based on the primary structure of the candidate enzyme and the codon preference of *E. coli*, and cloned into the pGEX-6p-1 expression vector with restriction enzymes *Bam*H I and *Xho* I. Recombinant plasmids were introduced into *E. coli* BL21 cells via the heat shock method. Transformants were selected on lysogeny broth (LB) agar plates containing ampicillin and cultured in liquid LB medium at 37°C with shaking at 140 rpm. When the culture reached an optical density at 600 nm of >0.8, protein expression was induced with 0.4 mM isopropyl β-D-1-thiogalactopyranoside at 16°C and 100 rpm for 12 hours. Following induction, cells were harvested by centrifugation at 8,000 rpm for 6 minutes at 4°C, and the cell pellet was resuspended in phosphate-buffered saline (PBS, pH 7.3–7.4). Cell disruption was performed using an ultrasonic homogenizer at 400 W for 10 minutes. The lysate was then centrifuged at 13,000 × *g* for 30 minutes at 4°C, and the resulting supernatant was incubated with GSTSep Glutathione Agarose Resin for 4–6 hours. After extensive washing to remove non-specific proteins, PreScission protease was added directly to the resin for on-column cleavage at 4°C for 12 hours. The eluate was collected and analyzed by sodium dodecyl sulfate-polyacrylamide gel electrophoresis (SDS-PAGE), and protein concentrations were determined using the BCA assay kit. The absorbance at 562 nm was measured using a microplate reader, and the protein concentration of each sample was calculated based on the standard curve and the sample volume used.

### Enzyme assays

Spectrophotometric assays were conducted in 2 mL quartz cuvettes with a 0.5 cm optical path length. The reaction buffer, coenzyme, and substrate were sequentially added, and the mixture was gently mixed. Absorbance changes at 340 nm were recorded, and the corresponding substrate concentration changes were calculated based on the molar extinction coefficient of the reduced coenzyme. In substrate screening assays, both the coenzyme and the substrate were used at a final concentration of 0.5 mM. The amount of enzyme added ranged from 2 to 10 μL, depending on the enzyme stock concentration. To assess substrate specificity, the catalytic activities of newly expressed candidate enzymes were tested against (*R*)-3-quinuclidinol, (*S*)-3-quinuclidinol, taurochenodeoxycholic acid (TCDCA), tauroursodeoxycholic acid (TUDCA), glucose, 20-hydroxyecdysone, ethanol, and β-estradiol. In addition, coenzyme preference (NAD^+^ or NADP^+^) was evaluated during suspected-substrate activity assays. The effects of various ions, including K^+^, Na^+^, Mg²^+^, Mn²^+^, Cu²^+^, and ethylenediaminetetraacetic acid (EDTA), on enzyme activity were subsequently investigated at different concentrations. This study investigated the enzymatic activity at different temperatures, ranging from 25°C to 95°C in 5°C increments, using a 50 mM PBS buffer. The reaction buffer and cuvettes were pre-incubated at different temperatures using a constant temperature water bath. After rapidly mixing the reaction system, the absorbance change at 340 nm was measured over 20–30 seconds. The initial reaction rate was calculated using the standard curve of coenzyme concentration versus absorbance for the reduced form. Additionally, the effect of the pH of the reaction mixture on enzyme activity was evaluated. PBS buffers (50 mM) with pH values ranging from 7.0 to 8.5 and Gly-NaOH buffers (50 mM) with pH values ranging from 9.0 to 10.0 were prepared. The enzyme’s initial reaction rates in these buffers were determined, and the activity change curve was plotted. Enzyme kinetic parameters (*K*_m_ and *k*_cat_) were determined by fitting the data to Lineweaver–Burk plots. All experiments were independently repeated at least three times.

### Three-dimensional structure prediction

The three-dimensional structure of the target protein was predicted using AlphaFold2, a deep learning framework developed by DeepMind. The amino acid sequence was provided as input, and a multiple sequence alignment was automatically generated against large sequence databases to identify evolutionary relationships. The model incorporated co-evolutionary signals and spatial constraints via a transformer-based architecture, resulting in a high-confidence structural prediction. The predicted models were ranked by per-residue confidence scores, and the top-ranked structure was selected for downstream analyses.

### Molecular dynamics simulations

AmberTools24 was downloaded from the official AMBER website (https://ambermd.org/) and utilized to prepare the system for molecular dynamics (MD) simulations (22). The preparation workflow began by launching the LEaP module, followed by loading the ff19SB and gaff2 force fields for protein parameterization. The three-dimensional structure of ScQR was then imported, and counterions (Na^+^ or Cl^−^) were added to neutralize the overall system charge. The solvated environment was established by immersing the system in a TIP3P water box using the solvateBox command. Upon verification of the molecular topology and system integrity, the topology and coordinate files required for simulation were generated. This study uses ParmEd, a Python package for handling molecular dynamics simulation parameters and topology files. ParmEd is capable of directly converting AMBER files (prmtop and inpcrd files) generated by tleap into GROMACS files. All subsequent molecular dynamics simulations and trajectory analyses were performed using GROMACS2022 (23). Energy minimization of the solvent, solute, side chains, and the full molecular system was carried out using a two-step protocol that combined the steepest descent algorithm followed by the conjugate gradient approach to eliminate unfavorable contacts and optimize the initial geometry. Subsequently, a multi-stage heating process was applied; the system temperatures were increased from 0 K to 300 K, 330 K, 360 K, and finally 390 K, respectively, over a period of 50 ps. The molecular dynamics simulations were conducted under canonical and isothermal-isobaric ensembles in succession, enabling accurate modeling of temperature- and pressure-dependent behavior of the molecular environment during system equilibration. Finally, 100 ns simulations were performed, saving 1,000 trajectory snapshots. To assess the thermally induced structural deviations, the root mean square deviation (RMSD) of the protein backbone was computed over the course of the simulation, providing a quantitative measure of conformational drift under elevated temperatures. In parallel, the root mean square fluctuation (RMSF) was analyzed for each residue, offering insights into site-specific flexibility and local mobility changes. Comparative analysis of RMSF profiles enabled the identification of regions with heightened thermal sensitivity.

### Computational design of enzyme

Enzyme-computational design was carried out in a structure-guided manner using LigandMPNN to redesign the amino acid sequence of the target enzyme based on its enzyme–substrate complex structure (24). LigandMPNN applies graph neural networks to explicitly model enzyme–substrate interactions, enabling targeted optimization of residues within the binding pocket and its surrounding regions while strictly preserving the native three-dimensional protein architecture. The core design principle of this approach was to maintain catalytic function while enhancing binding affinity, thereby ensuring that enzymatic activity is retained while ligand recognition is strengthened. Structural fidelity of the redesigned variants was evaluated by superposition with the original protein structure using PyMOL 3.0. Variants exhibiting an RMSD of less than 2 Å were considered to adopt conformations highly similar to that of the original enzyme, supporting functional stability. Kinetic parameters of the redesigned enzymes, including the Michaelis constant (*K*_m_), turnover number (*k*_cat_), and catalytic efficiency (*k*_cat_/*K*_m_), were calculated using UniKP. Here, *K*_m_ reflects enzyme–substrate affinity, with lower values indicating stronger binding, whereas *k*_cat_/*K*_m_ serves as a comprehensive metric of catalytic efficiency (25).

## RESULTS

### Heterologous expression

**Fig 2:**
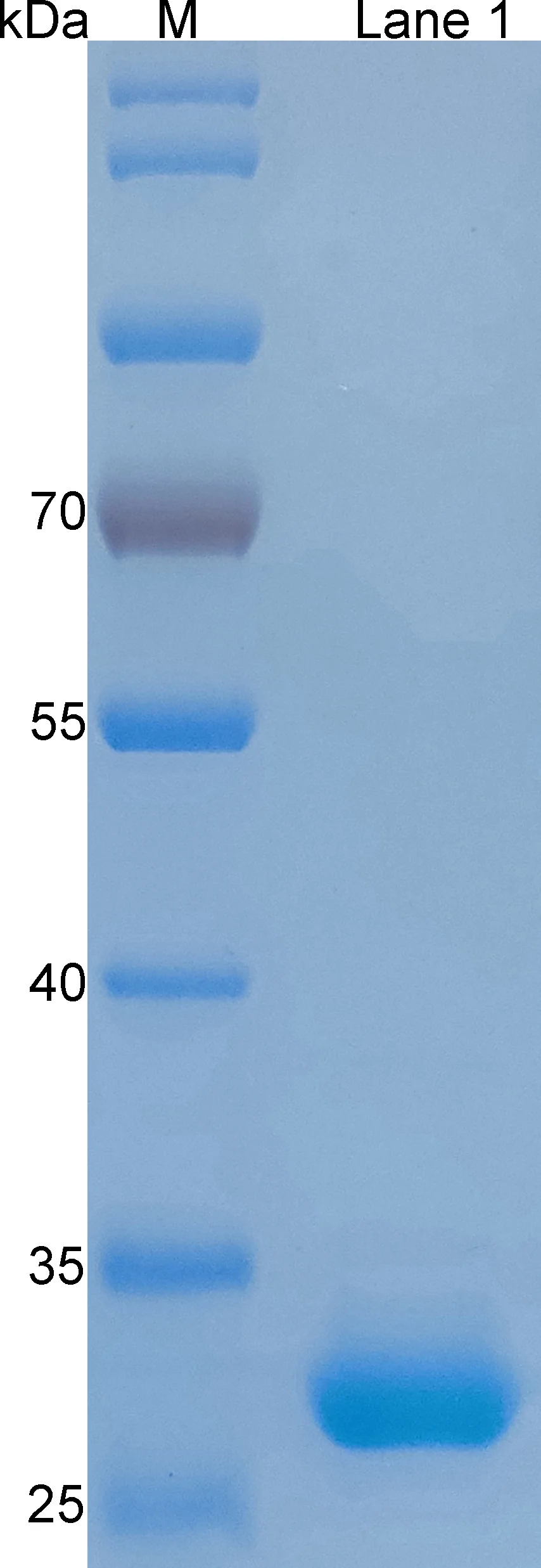
SDS-PAGE analysis of ScQR after cleavage of the N-terminal GST tag by PreScission protease. Lane M, protein ladder; lane 1, ScQR.

DNA sequencing of the recombinant plasmid confirmed the correct insertion of ScQR into the pGEX-6p-1 multiple cloning site, with *Bam*H I and *Xho* I restriction sites (accession number OM654425.1). Colony PCR was used to verify the recombinant plasmid constructs. The protein sequence, as encoded, was verified to be correct. ScQR consists of 259 amino acids and is registered in the NCBI database, with a theoretical molecular weight of 28.14 kDa. The expression system used in this study involved 500 mL of culture medium with recombinant *E. coli* BL21. The PreScission protease cleavage system was 2 mL, and the final enzyme concentration was 1.16 mg/mL, yielding a total of 2.32 mg of enzyme protein, which was used for subsequent experiments. The molecular weight distribution of the free enzyme, following removal of the N-terminal GST tag by PreScission protease, was analyzed by SDS-PAGE, and the observed results were consistent with the theoretical value (Fig. 2).

### Sequence and structure comparison

**Fig 3:**
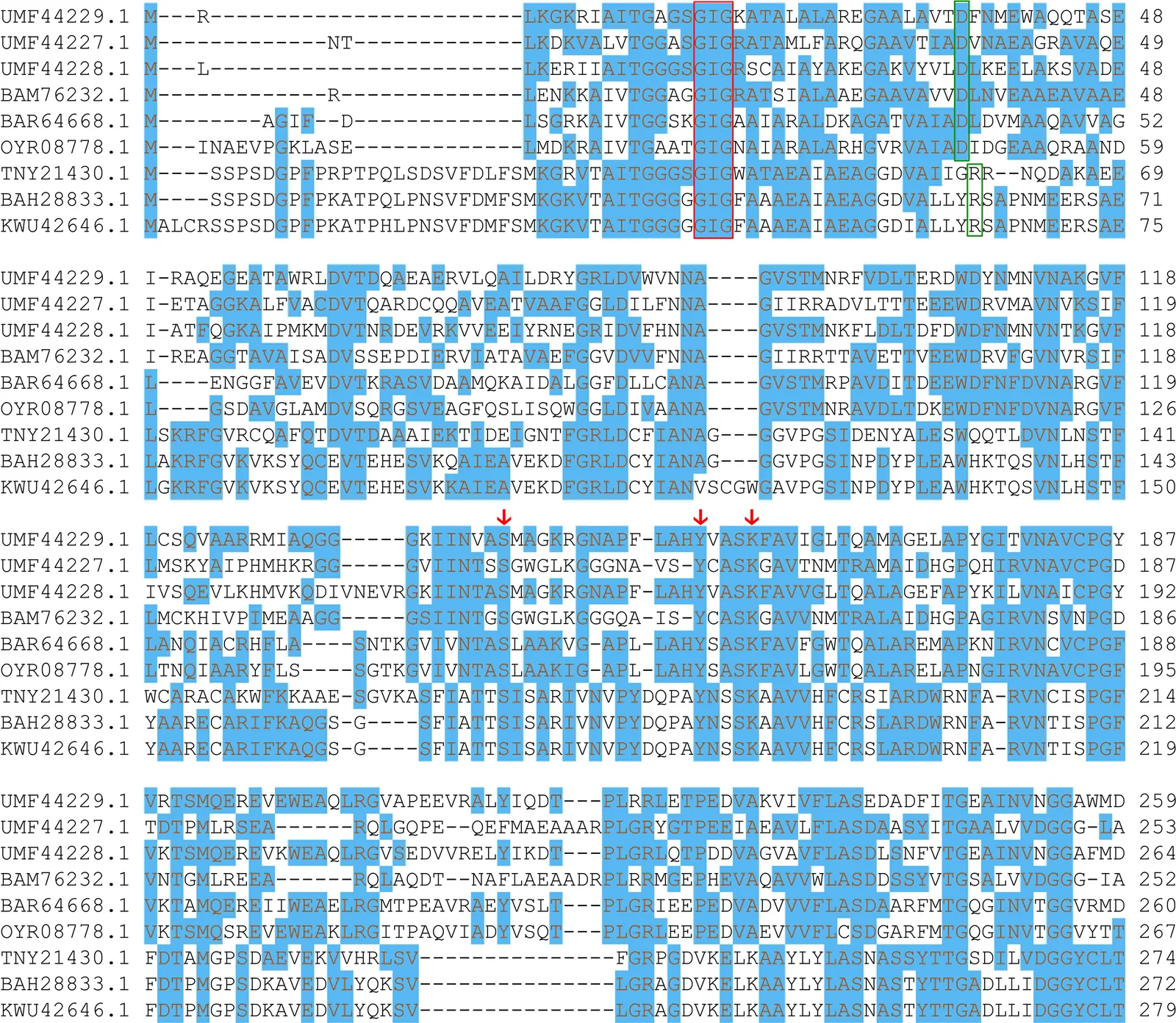
Multiple sequence alignments of ScQR (accession number UMF44229.1) and QRs from metagenomes (accession numbers UMF44227.1 and UMF44228.1), *Microbacterium luteolum* (accession number BAM76232.1), *Agrobacterium tumefaciens* (accession number BAR64668.1), *Ochrobactrum grignonense* (accession number OYR08778.1), *Rhodotorula diobovata* (accession number TNY21430.1), *Rhodotorula mucilaginosa* (accession number BAH28833.1), and *Rhodotorula* sp. JG-1b (accession number KWU42646.1). The red frames highlight the GIG motif; the red arrows indicate the catalytic triad (S-Y-K); and the green frames highlight the D or R residues, where the specific residue reflects the coenzyme preference, NAD(H) and NADP(H), respectively.

Figure 3 illustrates nine QRs, with the first three derived from hot environments. Although the percent identity of these three QRs ranges from 27.5% to 54.9% when compared to the six from mesophilic environments, they exhibit highly conserved motifs characteristic of the SDR superfamily. These include the GIG motif at the N-terminus, the catalytic triad (S-Y-K), and the coenzyme preference site, which for ScQR is S141-Y155-K159. The presence of D36 further suggests that ScQR is NAD(H) dependent.

**Fig 4:**
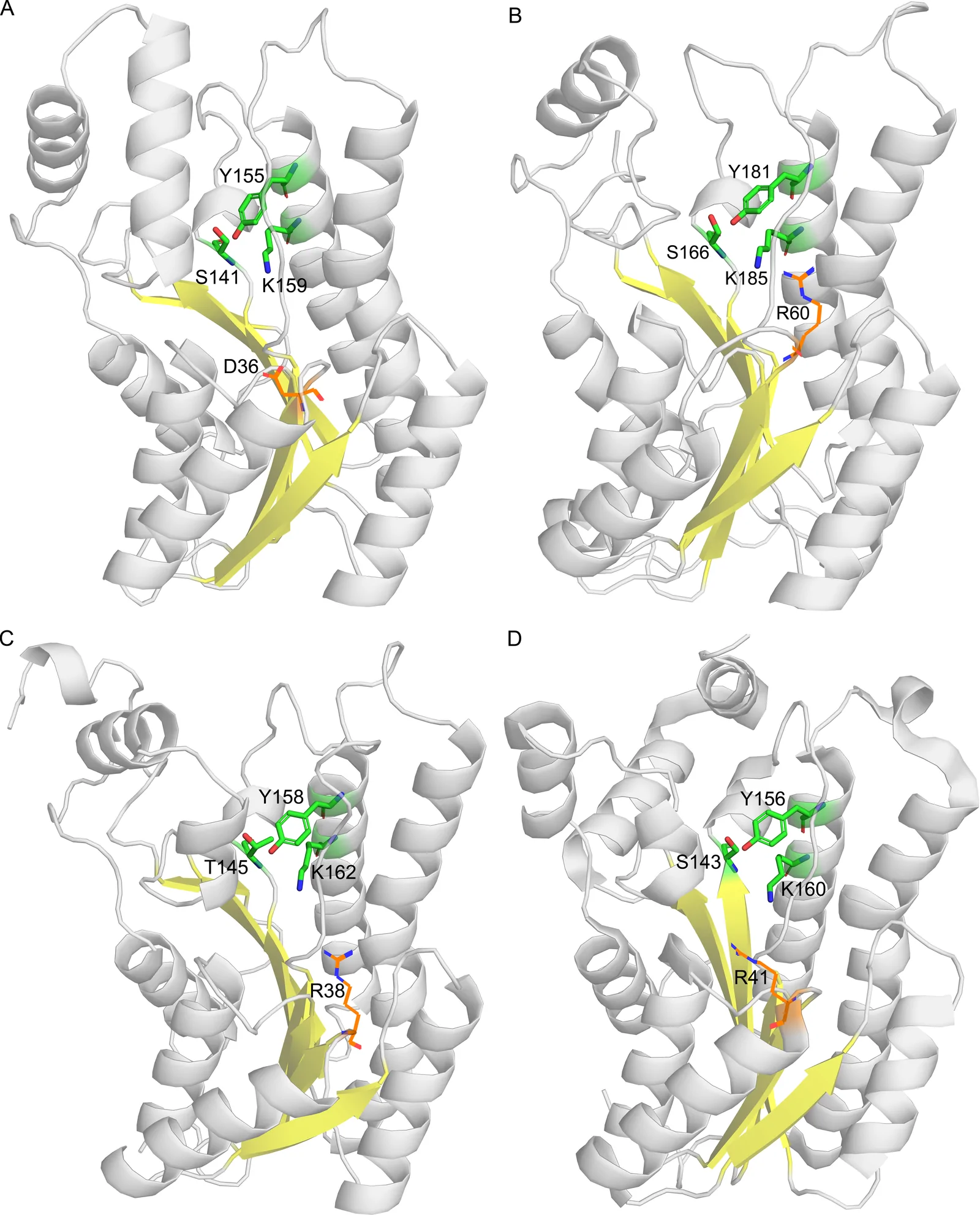
Comparison of the 3D structure of ScQR with three other members of the SDR family. (**A**) ScQR predicted using AlphaFold2, (**B**) *Rhodotorula mucilaginosa* QR (PDB 4O0L), (**C**) 7α-hydroxysteroid dehydrogenase from *Clostridium sardiniense* (PDB 5EPO), and (**D**) 7β-hydroxysteroid dehydrogenase from *Collinsella aerofaciens* (PDB 5GT9). The β-sheet structures are shown in yellow; the conserved catalytic triad (S-Y-K) is represented in the stick model, and the coenzyme-binding sites (D/R) are shown in the line model.

The three-dimensional structure of ScQR was predicted using AlphaFold2 (Fig. 4), revealing a canonical SDR architecture with a characteristic Rossmann-like fold. ScQR adopts a compact, globular framework centered on a core of seven parallel β-strands that assemble into a tightly packed β-sheet, which is enveloped by surrounding α-helices and connected by loop regions that collectively sculpt the overall fold (Fig. 4A). This topology is fully consistent with the conserved structural blueprint of SDR enzymes and provides a robust basis for interpreting the catalytic properties of ScQR. Notably, ScQR contains the hallmark catalytic triad of the SDR superfamily (S141-Y155-K159), supporting a classical proton-relay mechanism in which the tyrosine–lysine pair facilitates proton transfer and activation of the substrate hydroxyl group during hydride transfer. In addition, the presence of Asp36 is indicative of NAD(H) preference, in agreement with established cofactor specificity determinants that discriminate NAD(H)-dependent SDRs from their NADP(H)-utilizing counterparts. To place ScQR within the broader structural landscape of the SDR family, we performed comparative structural alignments with representative homologs, including quinuclidinone reductase from *Rhodotorula mucilaginosa* (PDB 4O0L), 7α-hydroxysteroid dehydrogenase from *Clostridium sardiniense* (PDB 5EPO), and 7β-hydroxysteroid dehydrogenase from *Collinsella aerofaciens* (PDB 5GT9) (26). These analyses yielded TM scores of 0.76–0.90, underscoring a high degree of structural conservation across these enzymes.

### Enzyme activity

The soluble GST-tagged ScQR was purified by affinity chromatography, followed by tag cleavage. All substrates mentioned in Materials and Methods, including (*R*)-3-quinuclidinol, (*S*)-3-quinuclidinol, TCDCA, TUDCA, glucose, 20-hydroxyecdysone, ethanol, and β-estradiol, were tested for activity with either NAD^+^ or NADP^+^ separately. The concentration of the substrate solutions and the measurement method were consistent across all tests, with absorbance changes measured at 340 nm. The final results indicate that the substrate for the newly discovered enzyme in this study is (*R*)-3-quinuclidinol. Notably, ScQR showed strict stereoselectivity, with no activity against (*S*)-3-quinuclidinol. Furthermore, experimental results indicated that the enzyme is NAD(H) dependent. The kinetic parameters were determined as follows: *K*_m_ = 0.236 ± 0.004 mM, *k*_cat_ = 0.762 ± 0.016 s⁻¹, and a calculated catalytic efficiency (*k*_cat_/*K*_m_) of 3.232 ± 0.087 s⁻¹ mM⁻¹.

### Optimum pH, temperature, and thermostability

**Fig 5:**
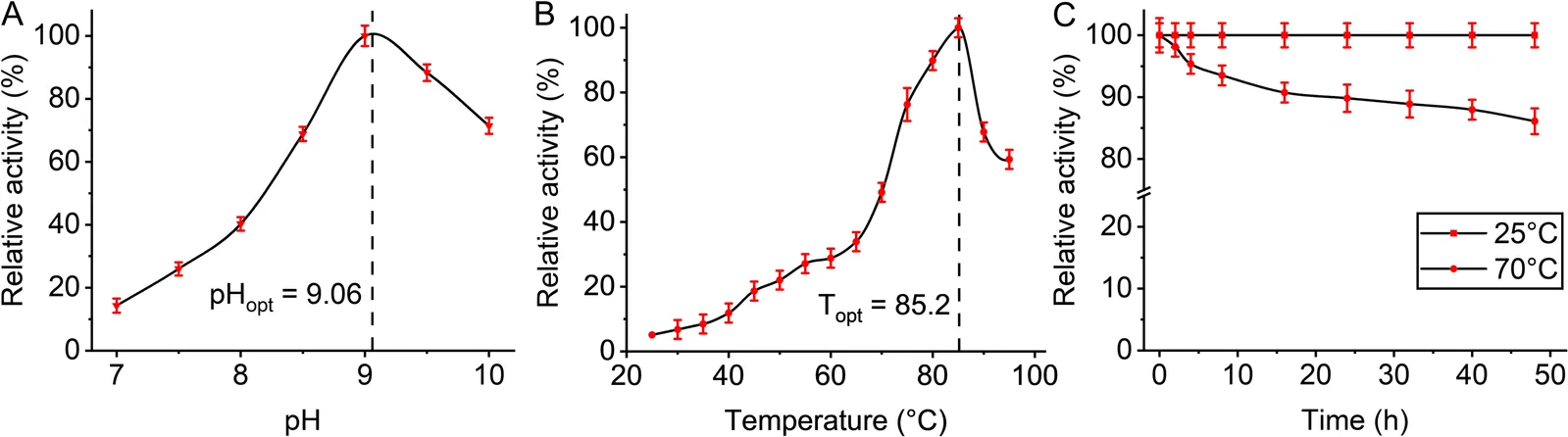
Determination of the optimal pH, temperature, and thermostability for ScQR. (**A**) The variation in ScQR activity within the pH range of 7–10 (with 0.5 pH unit intervals). (**B**) The change in enzyme activity within the temperature range of 25°C–95°C (with 5°C intervals). (**C**) Thermal stability of ScQR. The enzyme was incubated at 25°C and 70°C, respectively, with activity measurements taken at specified time intervals (0, 2, 4, 8, 16, 24, 32, 40, and 48 hours) to assess thermal stability based on relative activity. Error bars represent the standard deviation from at least three independent replicates.

The results of the determination of optimal pH and temperature (Fig. 5A and B) indicate that ScQR exhibits the highest activity in a reaction buffer at pH 9. Furthermore, the enzyme shows peak activity at approximately 85°C, suggesting that ScQR is a thermophilic enzyme. The purified ScQR was stored in sterile PBS (pH 7.5) after BCA and SDS-PAGE analysis. It was then incubated at 25°C and 70°C, with activity changes monitored over time (Fig. 5C). After 48 hours at 25°C, no change in activity was observed. At 70°C, after 48 hours, the enzyme retained 86% of its activity, demonstrating its exceptional thermal stability.

### Impact of ions on enzyme activity

**Fig 6:**
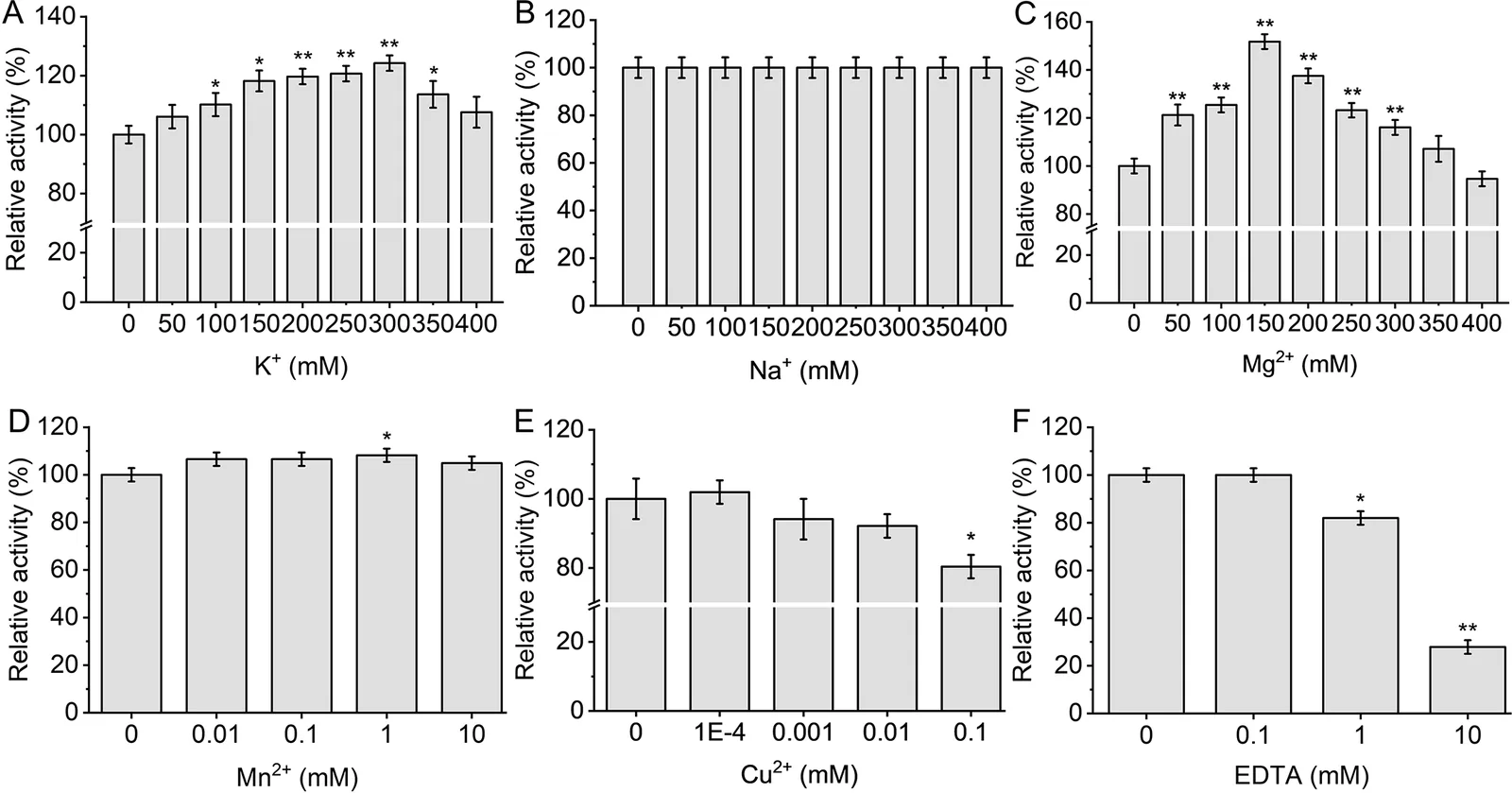
Impact of five metal ions and EDTA on ScQR activity. Enzyme activity was assessed in the presence of varying concentrations of K^+^ (**A**), Na^+^ (**B**), Mg²^+^ (**C**), Mn²^+^ (**D**), Cu²^+^ (**E**), and EDTA (**F**) added to the reaction buffer. Error bars indicate standard deviations based on a minimum of three replicates (*P < 0.05, **P < 0.001; compare with the 0 mM group).

In this study, the effects of different concentrations of metal ion hydrochloride salts (K^+^, Na^+^, Mg²^+^, Mn²^+^, and Cu²^+^), as well as EDTA, on ScQR activity were investigated (Fig. 6). K^+^ and Mg²^+^ exhibited the most significant enhancement of enzyme activity, with optimal increases of approximately 20% and 50% observed at 300 and 150 mM, respectively. Notably, Na^+^ and Mn²^+^ had minimal effects on activity within the tested concentration ranges, while Cu²^+^ and EDTA exhibited only inhibitory effects. EDTA, a metal-chelating agent, likely inhibited ScQR activity by sequestering essential metal cofactors.

### Mechanism of enzymatic inactivity

**Fig 7:**
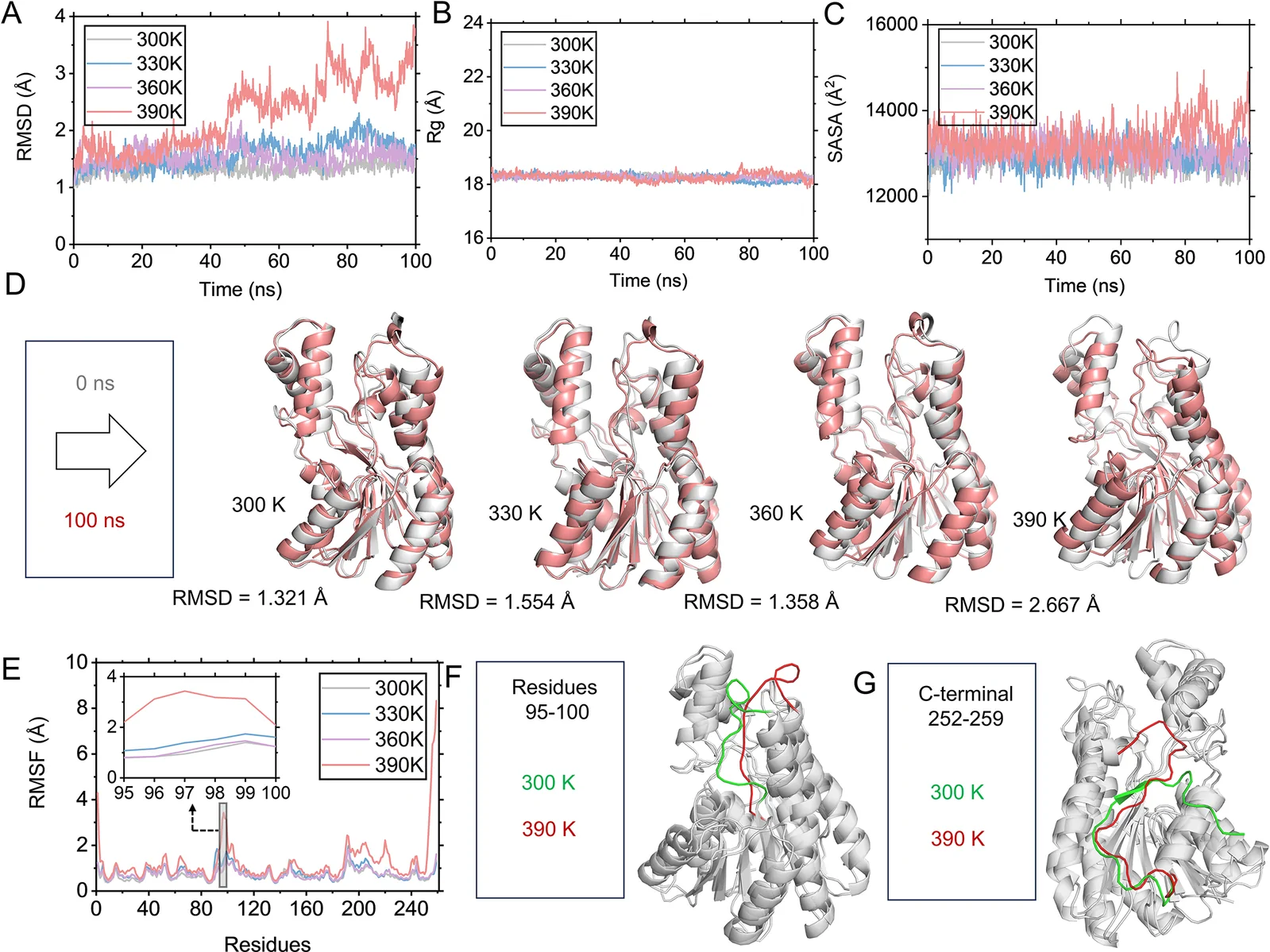
Structural stability under different temperatures was evaluated using 100 ns molecular dynamics simulations. (**A**) Root mean square deviation (RMSD) of the protein backbone atoms. (**B**) Changes in radius of gyration (*R*_g_). (**C**) Changes in solvent-accessible surface area (SASA). (**D**) Comparison of initial and final structures at different temperatures. (**E**) Root mean square fluctuation (RMSF) of all residues. (**F**) Residues 95–100; structural changes in ScQR stability at 300 K (colored in red) and 390 K (colored in green). (**G**) C-terminal (residues 252–259).

This study analyzed the molecular structural stability of ScQR at different temperatures through 100 ns MD simulations. The RMSD results (Fig. 7A) indicate that the enzyme structure remains stable at 300 K, 330 K, and 360 K, but when the system temperature increases to 390 K, the overall molecular structure fails to maintain stability. Consistently, the radius of gyration (*R*_g_) and solvent-accessible surface area profiles from molecular dynamics simulations also confirmed the relative thermal instability at 390 K (Fig. 7B and C). Further conformational analysis revealed a substantial structural deviation reaching 2.667 Å at 390 K (Fig. 7D). The RMSF results (Fig. 7E) show that the C-terminal region (residues 252–259) experiences increased flexibility and structural instability. Additionally, instability in residues 95–100 also becomes significantly higher. These results suggest that as the environmental temperature increases, the loss of the native ScQR molecular structure is likely due to destabilization at the carboxyl terminus and the 95–100 amino acid segment. In this study, the last molecular conformations from the MD simulations at 300 K and 390 K were compared (Fig. 7F and G).

### ScQR-directed evolution

**Fig 8:**
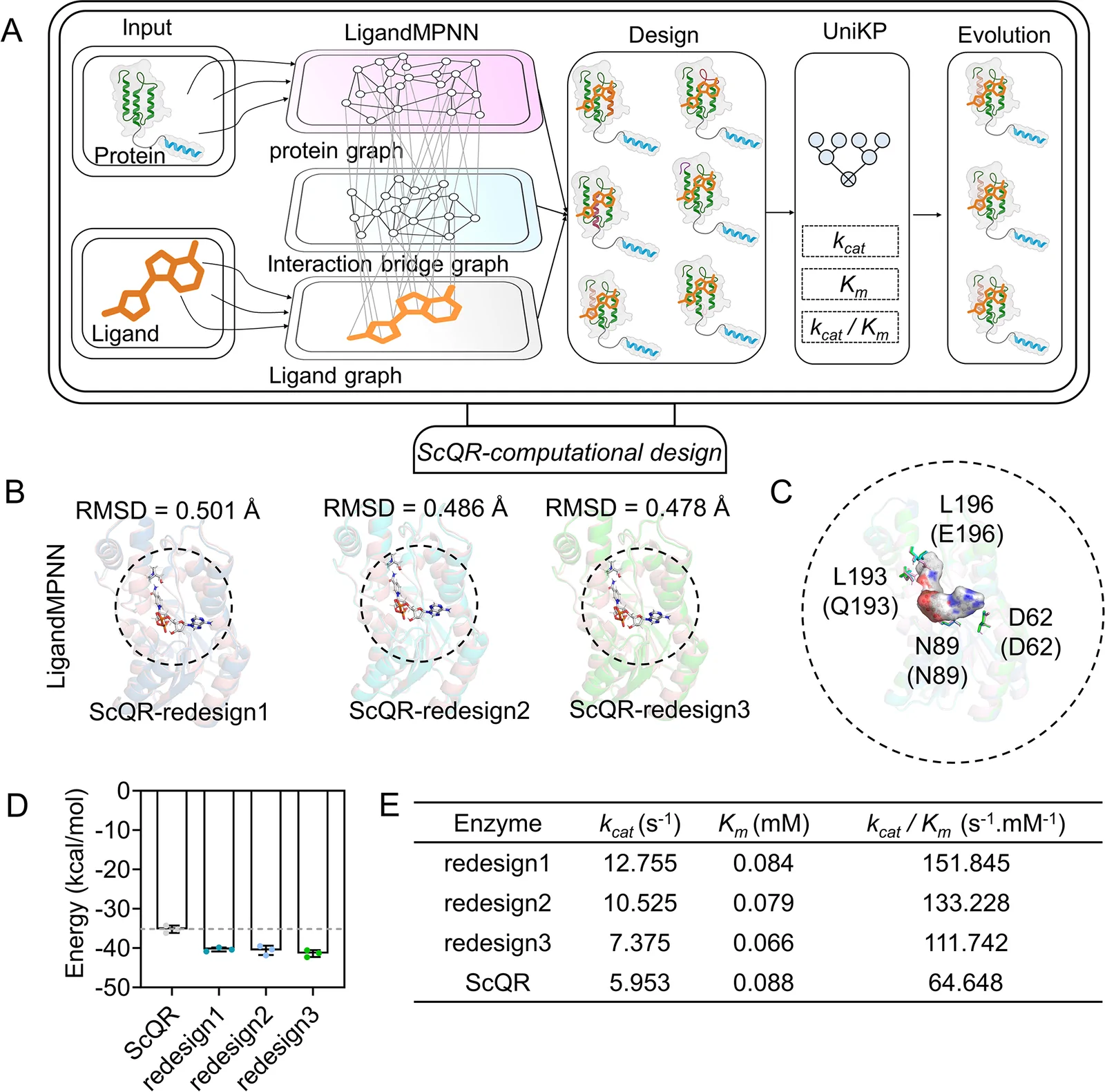
ScQR-computational design achieves high affinity and catalytic activity. (**A**) Workflow of enzyme-directed evolution based on artificial intelligence algorithms and structural information. (**B**) Structural superposition of ScQR and the evolved enzyme. (**C**) Key residues involved in substrate interactions in ScQR and the evolved enzyme. (**D**) Enzyme–substrate binding affinity calculated using the MM/GBSA method. (**E**) Enzyme kinetic parameters predicted based on UniKP.

Directed evolution mimics the processes of genetic mutation and natural selection observed in natural evolution, enabling the rapid optimization of enzymes. Based on the binding conformation of ScQR with its substrate, we employed a neural network trained on large-scale protein structural data sets to learn the relationships between sequence and structure, thereby achieving inverse design from a target structure to high-affinity sequences (Fig. 8A). Using UniKP to calculate enzyme kinetic parameters, we identified three enzyme variants exhibiting high substrate affinity: redesign1 (*K*_m_ = 0.084 mM, *k*_cat_ = 12.755 s⁻¹, and *k*_cat_/*K*_m_ = 151.845 s⁻¹ mM⁻¹); redesign2 (*K*_m_ = 0.079 mM, *k*_cat_ = 10.525 s⁻¹, and *k*_cat_/*K*_m_ = 133.228 s⁻¹ mM⁻¹); and redesign3 (*K*_m_ = 0.066 mM, *k*_cat_ = 7.375 s⁻¹, and *k*_cat_/*K*_m_ = 111.742 s⁻¹ mM⁻¹) (Fig. 8B through E). Three-dimensional models were constructed for the engineered sequences, and structural comparisons revealed RMSDs of 0.501, 0.486, and 0.478 Å relative to ScQR, indicating highly conserved overall conformations. Sequence alignment further demonstrated that L193 and L194 played critical roles during the directed evolution process (Fig. 8C). This structure-guided strategy—optimizing binding-site residues using LigandMPNN while preserving global protein stability—effectively enhances substrate affinity and catalytic efficiency, highlighting the potential of ScQR for biotechnological and pharmaceutical applications.

## DISCUSSION

Genomic analysis enables the identification of numerous ORFs from SDR superfamily members, such as the discovery of SDR members in *Bombyx mori* (27). Similarly, metagenomic analysis can reveal SDR superfamily members from specific environments (28, 29). However, confirming the precise functions (specificity and catalytic properties) of these SDR members often requires extensive follow-up experimental screening. Gene function predictions cannot directly determine the types of substrates unless the newly discovered protein shares a sufficiently high sequence identity with experimentally validated homologs, despite the emergence of new tools and models (25, 30). Furthermore, homology among SDR superfamily members is generally low (typically 20%–30%) (6), and even between members with identical specificity, the homology remains relatively low. For instance, the percent identity between 7α-HSDH (accession number AET80685.1) and 7β-HSDH (accession number AET80684.1) from *Clostridium sardiniense* is only 17.8%, despite both sharing the same chemical and regiospecificity and being regulated by the same promoter (31). Therefore, for SDR superfamily members, the final confirmation of their catalytic functions must be experimentally validated. Although this study used mesophilic QR in a BLASTP search to select multiple candidate proteins with similar functions, the function of ScQR was ultimately confirmed through extensive substrate/cofactor screening experiments. As mentioned above, although the homology among SDR members is relatively low, members from archaea, bacteria, and eukaryotes share conserved primary structural features, including the GIG motif, the conserved catalytic triad, and a length of approximately 250 amino acid residues (28, 32, 33), which aid in the classification and functional prediction of new members. It is noteworthy that, although the percent identity is relatively low, the members possess highly conserved three-dimensional structure (Rossmann fold) (6). However, they exhibit a wide substrate diversity and play a role in numerous metabolic processes within living organisms. This diversity in substrates may arise from the composition of several specific regions (34).

**Fig 9:**
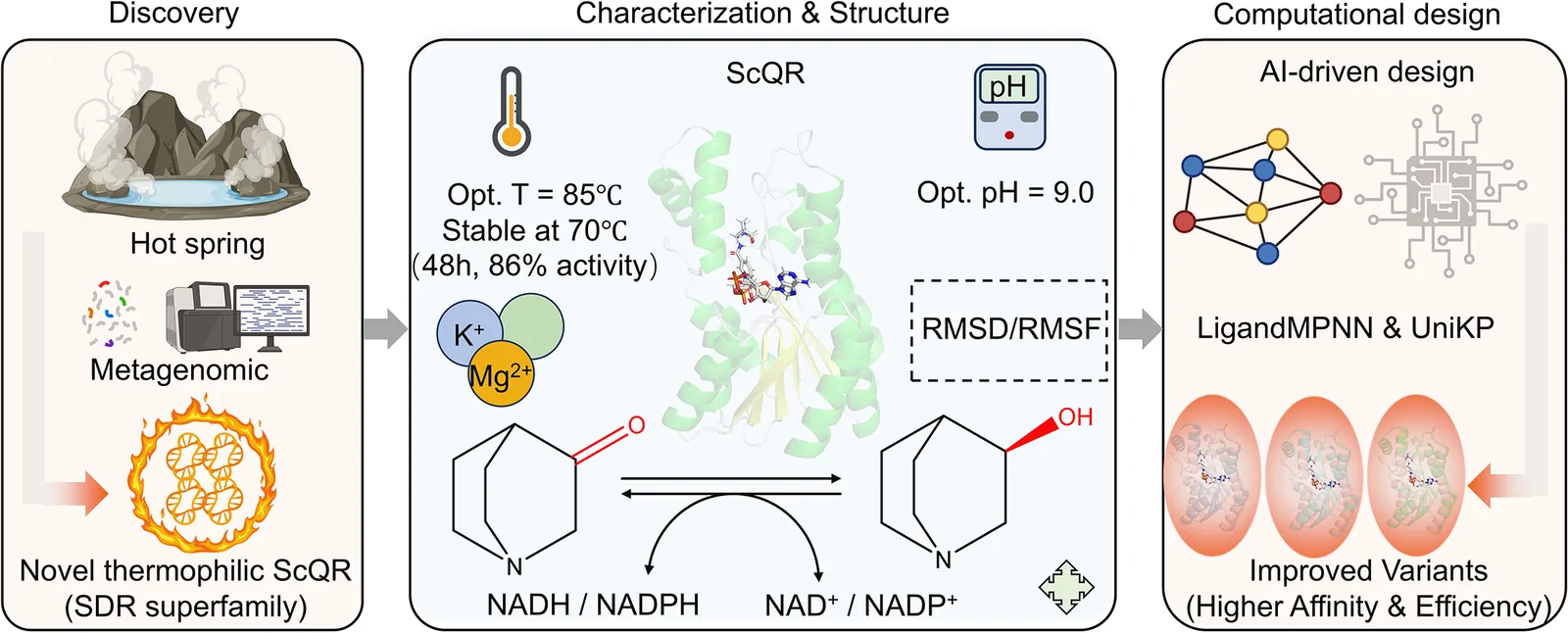
Overall schematic illustration of this study. Discovery, characterization, and structure-driven computational design of a novel 3-quinuclidinone reductase with exceptional thermal stability.

Archaea thrive in extreme environments on Earth, including those with extreme temperatures, pH, and pressures (35, 36). The enzymes they produce exhibit exceptional structural and functional stability under these conditions, making them valuable for industrial applications (4, 37). Moreover, archaea are linked to human diseases and therapeutic applications (38, 39). With the significant reduction in sequencing costs, metagenomics has become a powerful tool for discovering extremophilic enzymes (40). Over the past few decades, protein engineering has unraveled the structural basis of protein thermal stability, revealing multiple contributing factors (41–44). However, understanding the mechanisms behind protein thermal adaptation may require the integration of artificial intelligence (45, 46). Machine learning, in turn, enables the *de novo* design of novel enzymes (47, 48). In this study, molecular dynamics simulations revealed that ScQR undergoes structural disruption in the C-terminal and residues 95–100 regions at elevated temperatures. Building on these insights, we further achieved the directed evolution of ScQR by integrating state-of-the-art deep learning-based protein design and enzyme kinetics prediction frameworks, namely, LigandMPNN and UniKP. Specifically, protein residues and ligands were represented as a graph-based model in which interatomic distances and elemental features were used to encode molecular interactions, enabling a fine-grained simulation of biomolecular interactions within a biological environment (24). Through the incorporation of a two-layer neural network architecture, LigandMPNN facilitates efficient information propagation across the molecular graph, allowing precise capture of key interaction features such as hydrogen bonding and hydrophobic effects. Consequently, deep learning models such as LigandMPNN and UniKP play a distinctive and powerful role in the rational design of high-affinity enzymes for computational design. Notably, ScQR exhibits superior thermostability compared with other heat-resistant members of the SDR superfamily previously identified by our group (9). Taken together, these findings position ScQR as a highly promising biocatalyst for future biotechnological and pharmaceutical applications (Fig. 9).

### Conclusion

This study leveraged metagenomic analysis of thermal environments to identify a novel heat-resistant QR (ScQR) through homologous analysis, heterologous expression, and specificity screening. At 70°C, ScQR retained 86% of its activity after 48 hours, highlighting its remarkable thermal stability. Furthermore, K^+^ and Mg^2+^ significantly enhanced enzyme activity at specific concentrations. The industrial potential applications of ScQR include its involvement in the biosynthesis of drugs, such as revatropate, solifenacin, and talsaclidine, under high-temperature reaction conditions. Furthermore, this work highlights the synergistic integration of metagenomics and structure-driven computational design as a powerful strategy for the discovery and engineering of novel biocatalysts with distinctive and industrially valuable catalytic properties.
